# α-Latrotoxin Actions in the Absence of Extracellular Ca^2+^ Require Release of Stored Ca^2+^

**DOI:** 10.3390/toxins17020073

**Published:** 2025-02-06

**Authors:** Jennifer K. Blackburn, Quazi Sufia Islam, Ouafa Benlaouer, Svetlana A. Tonevitskaya, Evelina Petitto, Yuri A. Ushkaryov

**Affiliations:** 1 Medway School of Pharmacy, University of Kent, Chatham ME4 4TB, UK; jennifer.blackburn@yale.edu (J.K.B.); qsi@du.ac.bd (Q.S.I.); benlaouer.ouafa@gmail.com (O.B.); evelina.petitto@ashfiedmedcomms.com (E.P.); 2 Faculty of Biology and Biotechnology, HSE University, Moscow 117418, Russia; stonevitskaya@hse.ru

**Keywords:** α-Latrotoxin, calcium, intracellular Ca^2+^ stores, neurotransmitter release, neuromuscular junction, neuroblastoma cells, store-operated Ca^2+^ entry, ADGRL1, latrophilin-1

## Abstract

α-Latrotoxin (αLTX) causes exhaustive release of neurotransmitters from nerve terminals in the absence of extracellular Ca^2+^ (Ca^2+^_e_). To investigate the mechanisms underlying this effect, we loaded mouse neuromuscular junctions with BAPTA-AM. This membrane-permeable Ca^2+^-chelator demonstrates that Ca^2+^_e_-independent effects of αLTX require an increase in cytosolic Ca^2+^ (Ca^2+^_cyt_). We also show that thapsigargin, which depletes Ca^2+^ stores, induces neurotransmitter release, but inhibits the effect of αLTX. We then studied αLTX’s effects on Ca^2+^_cyt_ using neuroblastoma cells expressing signaling-capable or signaling-incapable variants of latrophilin-1, a G protein-coupled receptor of αLTX. Our results demonstrate that αLTX acts as a cation ionophore and a latrophilin agonist. In model cells at 0 Ca^2+^_e_, αLTX forms membrane pores and allows the influx of Na^+^; this reverses the Na^+^-Ca^2+^ exchanger, leading to the release of stored Ca^2+^ and inhibition of its extrusion. Concurrently, αLTX stimulates latrophilin signaling, which depletes a Ca^2+^ store and induces transient opening of Ca^2+^ channels in the plasmalemma that are sensitive to inhibitors of store-operated Ca^2+^ entry. These results indicate that Ca^2+^ release from intracellular stores and that Ca^2+^ influx through latrophilin-activated store-operated Ca^2+^ channels contributes to αLTX actions and may be involved in physiological control of neurotransmitter release at nerve terminals.

## 1. Introduction

α-Latrotoxin (αLTX) from black widow spider venom has been used for many years to study the complex mechanisms that underlie neurotransmitter release [1,2]. Soon after its first use as a molecular tool [3], the toxin’s complex activity was revealed [4,5] and, despite many examples of successful application of αLTX in neuronal studies [6,7,8,9], has so far remained enigmatic. The best-understood actions of αLTX include (i) its ability to form large, cation-permeable pores in cell membranes and (ii) the activation of the toxin’s presynaptic receptor, the adhesion-class G protein-coupled receptor latrophilin 1 (LPHN1, or ADGRL1 in the new nomenclature) [10]. These two actions occur concurrently but induce distinct patterns of neurotransmitter release [11]. However, the exact mechanisms underlying αLTX actions remain unclear. Particularly puzzling is the ability of αLTX to cause massive exocytosis of neurotransmitters in the absence of extracellular Ca^2+^ (Ca^2+^_e_) [1,5], which has led some researchers to propose that αLTX induces a novel pathway of exocytosis that operates independently of both Ca^2+^_cyt_ and the conventional vesicle fusion machinery [12].

αLTX is a large, tetrameric protein, with a propeller-like structure and mobile hydrophobic appendages at the base that facilitate its insertion into cell membranes [13]. At its center, αLTX has a large, non-selective, cation-permeable pore, which is 10 Å wide [14,15,16]. Thus, αLTX was thought to trigger exocytosis by enabling an influx of Ca^2+^ into presynaptic terminals through the pore it creates [4]. This pore is also wide enough to permit non-vesicular leakage of cytosolic neurotransmitters [17,18], and this may be helped by imperfections in the two-dimensional crystals that αLTX tetramers can form within the membrane [13]. Yet, these actions either require Ca^2+^_e_ or have a non-exocytotic character.

αLTX also stimulates exocytosis by activating presynaptic receptors. αLTX binds to ADGRL1 in the absence of Ca^2+^ [8], and activates a Gαq/phospholipase C/inositol trisphosphate signaling cascade that causes mobilization of Ca^2+^ from the endoplasmic reticulum (ER) [19,20,21,22]. The subsequent increase in cytosolic [Ca^2+^] ([Ca^2+^]_cyt_) triggers vesicular exocytosis. This effect is most clearly demonstrated when αLTX pore formation is prevented (i) by blocking toxin pores with La^3+^ or (ii) by using the mutant toxin LTX^N4C^, which does not form pores [19]. Surprisingly, to trigger exocytosis, the ADGRL1 signaling cascade still requires Ca^2+^_e_ [18,20]. Depletion of Ca^2+^ stores is known to activate Stromal interaction molecule (STIM) proteins [23,24,25,26,27], which trigger the opening of store-operated Ca^2+^ channels (SOCCs) [28,29,30] and induce store-operated Ca^2+^_e_ entry (SOCE) into cells. In support of a possible contribution of these channels to ADGRL1-mediated αLTX action, it can be blocked by SOCC inhibitors [20].

αLTX has been reported to release Ca^2+^ from intracellular stores even in the absence of Ca^2+^_e_. Tsang et al. [31] found that at frog nerve terminals, in Ca^2+^-free conditions, αLTX releases Ca^2+^ from mitochondria (MC), which depends on Na^+^ influx. Such a mechanism of Ca^2+^ release could include the reversal of Na^+^/Ca^2+^ exchanger (NCX) [32,33], one of the major Ca^2+^ extrusion pumps located on the plasma membrane, ER, and MC membranes [34,35,36,37,38]. Surprisingly, in this work, the Ca^2+^_e_-independent release of stored Ca^2+^ apparently had no effect on acetylcholine (ACh) exocytosis induced by αLTX [31]. Thus, the role of Ca^2+^_cyt_ in the Ca^2+^_e_-independent actions of αLTX remains controversial.

In this paper, we investigate the pore-dependent and receptor-dependent effects of αLTX on Ca^2+^ release and Ca^2+^ influx, and the role of Ca^2+^_cyt_ in αLTX-induced exocytosis. First, we demonstrate that Ca^2+^_cyt_ is required for αLTX-evoked secretion of ACh at mouse neuromuscular junctions (NMJ) in the absence of Ca^2+^_e_. Then, using signaling and a non-signaling ADGRL1 construct, we show that the αLTX pore and ADGRL1 signaling contribute to Ca^2+^ release, but target distinct Ca^2+^ stores. We discuss the role of SOCCs and Ca^2+^ extrusion in αLTX-induced elevation of Ca^2+^_cyt_ levels.

## 2. Results

### 2.1. α. LTX-Induced Release of Neurotransmitter Requires Intracellular Ca^2+^

**Figure 1:**
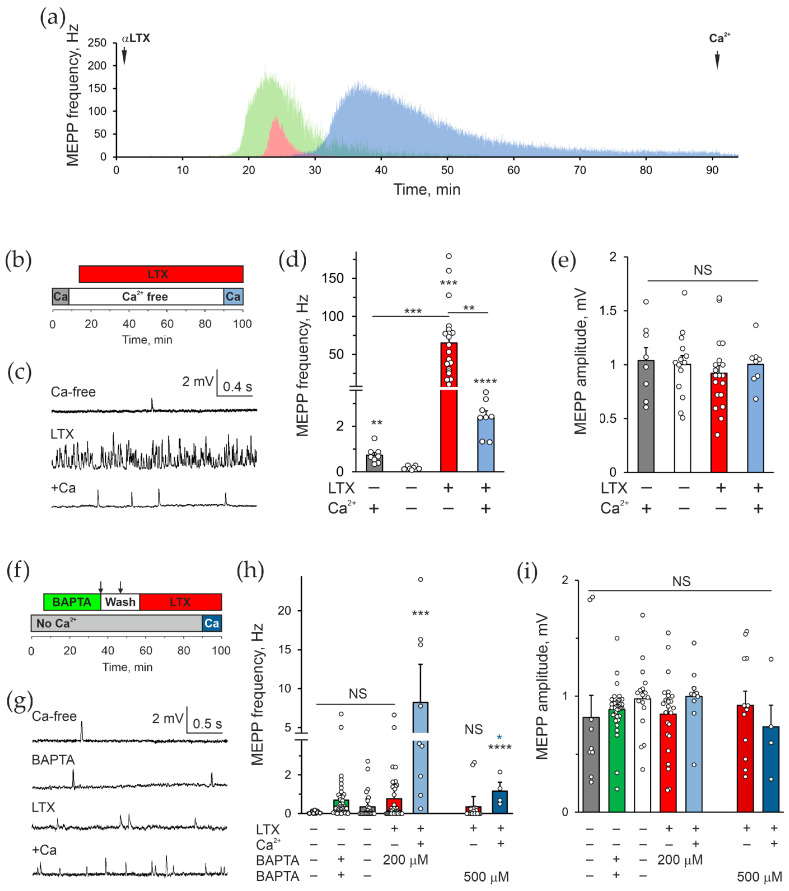
αLTX actions do not require extracellular Ca^2+^ but strictly depend on intracellular Ca^2+^. (**a**) Examples of the effect of 0.5 nM αLTX on the frequency of spontaneous MEPPs in mouse neuromuscular preparations in the absence of Ca^2+^_e_, continuously recorded from individual muscle fibers. (**b**) The control experimental protocol: initial incubation with 2 mM Ca^2+^_e_; removal of Ca^2+^_e_; addition of 0.5 nM αLTX; reintroduction of 2 mM Ca^2+^_e_. (**c**) Representative *V*m recordings during respective experimental stages. (**d**,**e**) Mean MEPP frequencies and amplitudes during the experimental stages as indicated below. (**f**) The Ca^2+^_cyt_ chelation protocol: initial incubation in a Ca^2+^_e_-free buffer; incubation with 200–500 μM BAPTA-AM; two extended washing steps with a Ca^2+^-free buffer; addition of 0.5 nM αLTX; reintroduction of 2 mM Ca^2+^_e_. (**g**) Representative *V*m recordings under the experimental conditions indicated. (**h**,**i**) Mean MEPP frequencies and amplitudes during respective experimental stages. The bars are the means ± *SEM*; the bar colors correspond to protocol phases; the underlying data points are shown as white circles; asterisks show statistical significance compared to Ca^2+^_e_-free control, unless indicated by lines; the blue asterisk in (**h**) compares the values indicated by the two blue bars; *, *p* < 0.05; **, *p* < 0.01; ***, *p* < 0.001; ****, *p* < 0.0001; *NS*, non-significant; for each condition shown, *n* = 9–43 individual muscle fibers, from 3 to 4 independent neuromuscular preparations.

To study the role of intracellular Ca^2+^ in the effect of αLTX on neurotransmitter release in the absence of Ca^2+^_e_, we used mouse neuromuscular preparations that are known to respond to αLTX by massive exocytosis of synaptic vesicles both in the presence and absence of Ca^2+^_e_ [11,39,40]. In a Ca^2+^-free medium, 0.1–0.5 nM αLTX caused a dramatic (up to 1500-fold) increase in the frequency of miniature end-plate potentials (MEPPs), which correspond to “spontaneous” fusion events of synaptic vesicles containing ACh (Figure 1a,c,d). This rise in MEPP frequency was very gradual, only occurring 15–30 min after α-LTX addition and continuing for 10–70 min, with the frequency gradually decreasing until no MEPPs could be detected (Figure 1a). The average maximal MEPP frequency was 101 ± 13 Hz (*n* = 18), and on average 43.8 × 10^3^ ± 15 × 10^3^ vesicles were released from each continuously recorded nerve terminal (*n* = 10).

αLTX action was purely presynaptic because it only affected the frequency, but not the amplitude, of the MEPPs (Figure 1e). The recycling of synaptic vesicles in motor neuron terminals is inhibited in the absence of Ca^2+^_e_ [41,42], and indeed over time the toxin essentially depleted all releasable vesicles from nerve terminals, as evidenced by the failure of 2 mM Ca^2+^_e_ added after MEPP cessation to increase the frequency of exocytotic events (Figure 1a,c,d).

To probe the role of Ca^2+^_cyt_ in the αLTX-evoked exocytosis, we pretreated the preparation with a membranepermeable Ca^2+^ chelator, BAPTA-AM, whose excess was later washed out from the bath (Figure 1f). As was observed under the microscope, a substantial amount of 200 μM BAPTA-AM precipitated out of solution, potentially leading to a decreased final concentration of BAPTA inside the cells.

To assess the level of cytosolic BAPTA achieved in this experiment, we used 20 mM KCl, which depolarizes the nerve terminal membrane, resulting in the opening of voltage-gated Ca^2+^ channels, an influx of Ca^2+^_e_ and ACh exocytosis (Figure S1a). If the cytosolic level of BAPTA exceeded 100 μM, it would have chelated most Ca^2+^ entering nerve terminals and blocked exocytosis [43]. In control NMJs, 20 mM KCl/2 mM Ca^2+^_e_ triggered a long train of high-frequency exocytosis, resulting in the eventual release of all vesicles. This suggests that KCl affected not only readily releasable vesicles, but also reserve-pool vesicles, and thus acted by increasing the general [Ca^2+^]_cyt_. Pretreatment of neuromuscular preparations with 200 μM BAPTA-AM significantly inhibited the exocytotic response to 20 mM KCl/2 mM Ca^2+^ (Figure S1b,c). However, KCl still induced a substantial increase in the frequency of MEPPs (Figure S1c), indicating that the cytosolic concentration of BAPTA was not adequate to chelate all Ca^2+^_e_ entering nerve terminals.

We therefore conducted further experiments with 500 μM BAPTA-AM. Its addition initially caused some increase in MEPP frequency (Figure 1h). As the drug was hydrolyzed in the cytosol and regained the ability to chelate Ca^2+^, the average MEPP frequency fell to the level of the Ca^2+^-free control, where most cells showed no MEPPs, while few NMJs, located deeper in the muscle and thus less affected by BAPTA, appeared to show a higher MEPP frequency (Figure 1h). Subsequent application of αLTX caused a very small increase in MEPP frequency compared to its effect in non-BAPTA-treated terminals in the absence of Ca^2+^_e_ (Figure 1d,h). Again, the distribution of MEPP frequencies was clearly bimodal: most NMJs were completely “silent”, whereas the deeper terminals showed some exocytotic activity (Figure 1h). This inhibition of the toxin effect was not caused by the depletion of synaptic vesicles, because when 2 mM Ca^2+^ was added to the medium at the end of recording, it entered the cells via αLTX pores and induced a strong increase in MEPP frequency, with all NMJs displaying an elevated activity. With the increased concentration of BAPTA-AM applied, the number of active synapses and their exocytotic activity significantly decreased. We interpret these results as demonstrating two important aspects: (i) when BAPTA-AM is used to load cells, its cytosolic concentration is often much lower than expected due to precipitation and is possibly insufficient to chelate all Ca^2+^_cyt_; (ii) even in 0 Ca^2+^_e_, αLTX strictly requires Ca^2+^_cyt_ to induce release of ACh from motor neuron terminals.

### 2.2. α. LTX Induces Release of Ca^2+^ from Intracellular Stores

**Figure 2:**
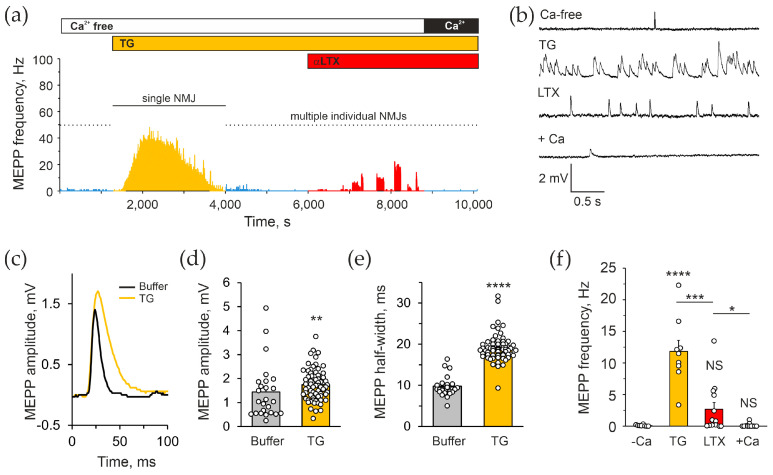
The depletion of intracellular Ca^2+^ stores inhibits the Ca^2+^_e_-independent actions of αLTX. (**a**) An example of the effect of TG and subsequent αLTX on the frequency of spontaneous MEPPs. The experimental protocol shown above the trace included the following phases: initial incubation in a Ca^2+^_e_-free buffer; addition of 10 μM TG; addition of 0.5 nM αLTX; and reintroduction of 2 mM Ca^2+^_e_. (**b**) Representative *V*m recordings during respective experimental stages. (**c**) Average MEPPs in the absence and presence of TG, in the absence of Ca^2+^_e_. (**d**,**e**) Mean MEPP amplitudes and half-widths during the indicated experimental stages. (**f**) Mean MEPP frequencies’ respective experimental stages. The bars are the means ± *SEM*; the bar colors correspond to protocol phases; asterisks show statistical significance compared to Ca^2+^_e_-free control; the underlying data points are shown as white circles; *, *p* < 0.05; **, *p* < 0.01; ***, *p* < 0.001; ****, *p* < 0.0001; *NS*, non-significant; *n* = 12–36 individual muscle fibers from 4 to 7 independent neuromuscular preparations.

The observation above suggested that αLTX might act by releasing Ca^2+^ from some intracellular Ca^2+^ stores. Given the fact that previous publications demonstrated the ability of thapsigargin (TG) to inhibit neurotransmitter exocytosis induced by a mutant toxin, LTX^N4C^ [20,44], we tested whether TG, an inhibitor of the sarcoplasmic–endoplasmic reticulum Ca^2+^ ATPase (SERCA), could also inhibit the action of the wild-type α-LTX. Application of 10 μM TG to mouse NMJs in the absence of Ca^2+^_e_ (Figure 2a), induced a strong increase in the frequency of MEPPs that gradually subsided to a lower-than-control level within 10–30 min (Figure 2a,b,f). The effect developed within 2–5 min, with MEPP frequencies reaching ~50 Hz at the peak and 11.8 ± 1.8 Hz on average. TG clearly acted both presynaptically (increasing MEPP frequency) and postsynaptically (raising the amplitude and duration of MEPPs) (Figure 2c–e). Nevertheless, the presynaptic action of TG allowed us to assess the role of presynaptic Ca^2+^ stores on the action of αLTX.

When αLTX was applied to neuromuscular preparations pretreated with TG for 30–60 min, the effect of αLTX was greatly reduced but not completely blocked, and with time disappeared entirely (Figure 2a,f). Furthermore, 2 mM Ca^2+^_e_ added at the end of TG/αLTX activity was unable to stimulate any new exocytotic activity (Figure 1a,f). This suggests that (i) part of αLTX action induces Ca^2+^ release from the ER that is sensitive to TG; however, (ii) the toxin also mobilizes Ca^2+^ from some other stores that are not TG-sensitive; and (iii) in Ca^2+^_e_-free conditions, both TG and αLTX block vesicle recycling and deplete the nerve terminals of all synaptic vesicles.

Thus, our experiments confirmed the critical role of stored Ca^2+^ in the effects of wild-type αLTX in the absence of Ca^2+^_e_.

### 2.3. ADGRL1 Expression in NB2a Cells

**Figure 3:**
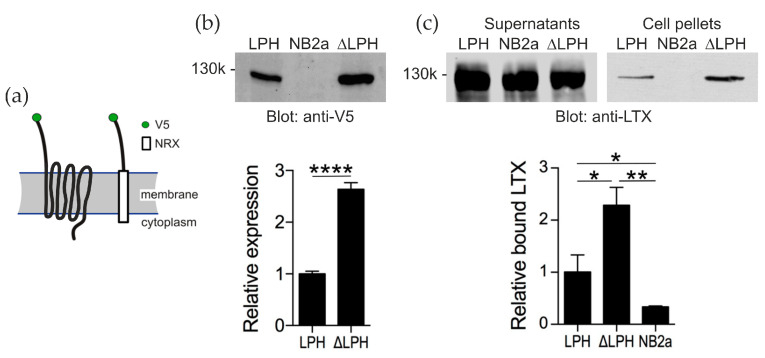
ADGRL1 constructs expressed in NB2a cells specifically bind αLTX. (**a**) The structures and membrane topologies of the two ADGRL1 constructs. (**b**) Stable expression of the ADGRL1 constructs in NB2a cells. Whole-cell lysates were separated by 8% SDS-PAGE, blotted and probed with an anti-V5 antibody. Top, a typical Western blot representative of four independent experiments. Bottom, quantification of the expression data; *n* = 4. NB2a, un-transfected cells. (**c**) The expressed ADGRL1 constructs bind αLTX. Transfected cells were incubated with 5 nM αLTX and centrifuged. The supernatants and cell pellets were separated by SDS-PAGE, blotted and probed with an anti-LTX antibody. Top, a representative Western blot showing unbound αLTX in the supernatants and receptor-bound αLTX in the cell pellets. Bottom, relative amounts of αLTX bound to respective cells; *n* = 3. *, *p* < 0.05; **, *p* < 0.01; ****, *p* < 0.0001.

To study the role of αLTX in [Ca^2+^]_cyt_ regulation independently of ADGRL1 signaling, two constructs were expressed in a murine neuroblastoma cell line (NB2a). A full-size ADGRL1 (LPH) and a truncated ADGRL1 construct (ΔLPH) were tagged with an extracellular V5 epitope for specific detection of expressed receptor (Figure 3a). In the signaling-incapable ΔLPH, the N-terminal domain of ADGRL1 (for αLTX binding) was fused to the transmembrane domain of neurexin I, necessary for cell-surface expression of the ADGRL1 extracellular domain but unable to mediate G protein signaling.

ΔLPH was expressed at a higher level (2.6 ± 0.1 times) than LPH (Figure 3b). To see if this difference in expression resulted in ΔLPH-expressing cells binding more αLTX than LPH-expressing cells, the cultures were incubated with an excessive concentration of αLTX (5 nM). ΔLPH bound 2.3 ± 0.4 times more αLTX than LPH (Figure 3c). These differences in expression level and αLTX interaction meant that subsequent [Ca^2+^]_cyt_ recordings had to be analyzed relative to a minimum and maximum Ca^2+^ indicator fluorescence.

### 2.4. α. LTX Mobilizes Intracellular Ca^2+^ Stores via Both Signaling and Non-Signaling Mechanisms

**Figure 4:**
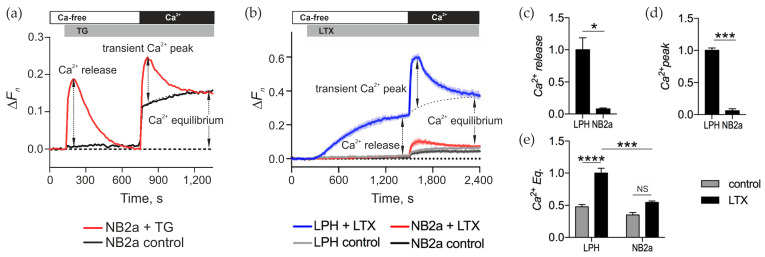
αLTX releases intracellular Ca^2+^ and triggers vast Ca^2+^ influx in cells expressing full-size receptors only. (**a**) A representative trace of changes in Ca^2+^ fluorescence in Fluo-4-loaded NB2a cells treated with 0.3 μM TG. The following characteristic stages are indicated: in the absence of Ca^2+^_e_, TG induces intracellular Ca^2+^ release; reintroduction of Ca^2+^_e_ leads to a transient Ca^2+^ influx (Ca^2+^ peak) and Ca^2+^_cyt_ equilibrium (Ca^2+^ Eq). (**b**) Representative fluorescence traces showing αLTX-mediated effects in control cells and cells expressing LPH. (**c**) Relative Ca^2+^ release measured at end of Ca^2+^_e_-free period. (**d**) Amplitude of transient Ca^2+^ peak on the addition of Ca^2+^_e_. (**e**) Relative Ca^2+^_cyt_ Eq in the presence of Ca^2+^_e_. Bars show the means ± *SEM* from 3 to 7 independent experiments performed in triplicate. *, *p* < 0.05; ***, *p* < 0.001; ****, *p* < 0.0001; *NS*, non-significant.

Mobilization of Ca^2+^ from intracellular stores was detected using a membrane-permeable Ca^2+^-sensing dye, Fluo-4 AM, which was loaded into the ADGRL1-transfected or control neuroblastoma cells, where the dye was hydrolyzed and entrapped in the cytosol. As a control stimulant for Ca^2+^ release we used TG. Application of this drug in 0 Ca^2+^_e_ allows one to clearly identify Ca^2+^ release from the ER (Figure 4a, Ca^2+^ release). Subsequent addition of 2 mM Ca^2+^ to the medium leads to a transient peak of Ca^2+^_e_ influx that reveals the brief opening of SOCCs (Figure 4a, Ca^2+^ peak). Finally, after the SOCCs close, a balance of Ca^2+^ influx and extrusion is achieved, termed here “Ca^2+^_cyt_ equilibrium” (Figure 4a, Ca^2+^ Eq).

To study the αLTX-induced effects on Ca^2+^ release from intracellular stores and influx of Ca^2+^_e_, NB2a cells (un-transfected or transfected with LPH) were loaded with Fluo-4 AM and stimulated in a three-step protocol. First, the cells were incubated in a Ca^2+^-free buffer. Then, αLTX and/or different pharmacological agents were added to detect their effects on Ca^2+^ release. Subsequently, the cells were exposed to 2 mM Ca^2+^_e_ to reveal changes in Ca^2+^ influx (Figure 4b). The changes in Fluo-4 fluorescence were continuously recorded as described in Section 5. In the Ca^2+^-free medium, αLTX caused release of intracellular Ca^2+^ only in ADGRL1-expressing cells (Figure 4b,c). This release occurred over several minutes, reaching a plateau which was apparently determined by the new equilibrium between [Ca^2+^]_cyt_ and Ca^2+^ extrusion mechanisms (Figure 4b). Upon the addition of Ca^2+^_e_, there was an immediate large influx of Ca^2+^, which soon decayed to a relatively high, new level (Ca^2+^ Eq). This transient opening of Ca^2+^-permeable channels was absent in control cells, while a small peak of Ca^2+^ influx sometimes appeared in αLTX-treated un-transfected cells due to a low non-specific binding of αLTX to cell membranes (Figure 4b,d). Also, the Ca^2+^ Eq in control cells was lower than in αLTX-treated cells (Figure 4b,e); this could involve several αLTX-mediated mechanisms: formation of toxin pores in the membrane, opening of some permanent Ca^2+^ channels and/or perturbation of Ca^2+^ extrusion.

**Figure 5:**
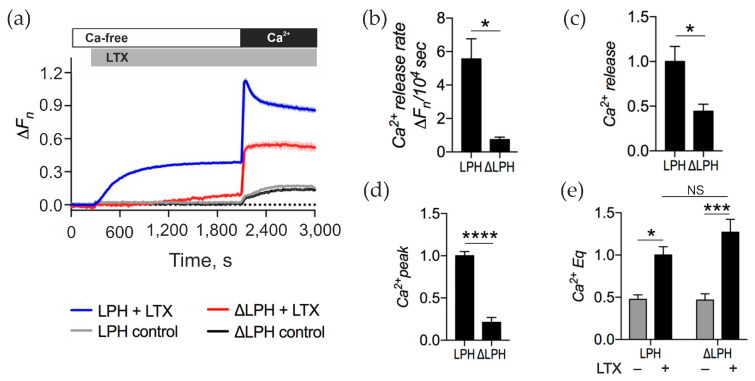
αLTX-mediated Ca^2+^_cyt_ regulation involves signaling and non-signaling mechanisms. (**a**) LPH- and ΔLPH-expressing NB2a cells were incubated in Ca^2+^-free buffer, then treated with 1 nM αLTX, and exposed to 2 mM Ca^2+^_e_. The fluorescence traces shown are the averages of three replicates and representative of four independent experiments. (**b**) Initial rate of intracellular Ca^2+^ release. (**c**) Relative Ca^2+^ release at the end of Ca^2+^_e_-free period. (**d**) Amplitude of the transient Ca^2+^ influx peak in the presence of Ca^2+^_e_. (**e**) Relative Ca^2+^ Eq in the presence of Ca^2+^_e_. Bars show the means ± *SEM*; *, *p* < 0.05; ***, *p* < 0.001; ****, *p* < 0.0001; *NS*, non-significant.

In cells expressing ΔLPH, αLTX-mediated Ca^2+^ release in the absence of Ca^2+^_e_ was strongly attenuated (Figure 5a). Also, the rate of this release was 7.5-fold slower (Figure 5b), and [Ca^2+^]_cyt_ continued to increase over the whole incubation period, whereas in LPH-expressing NB2a cells, a plateau of [Ca^2+^]_cyt_ was reached within ~10 min. After 30 min of αLTX stimulation, Ca^2+^ release in ΔLPH cells was about half of that in LPH cells (Figure 5c). Upon the addition of Ca^2+^_e_, αLTX failed to produce in ΔLPH cells the distinct transient Ca^2+^ peak that was seen in LPH cells (Figure 5d), demonstrating that ADGRL1 signaling leads to the opening of transient Ca^2+^ channels in the cell membrane. After the decay of the transient Ca^2+^ peak, [Ca^2+^]_cyt_ remained high and did not differ significantly between the ADGRL1 constructs (Figure 5e), which indicates that the maintenance of a very high Ca^2+^ Eq was due to αLTX only and did not involve any receptor-mediated signaling.

These results indicate that αLTX triggers Ca^2+^ release by two mechanisms. First, αLTX binds to and activates ADGRL1. Next, it inserts itself into the cell membrane and forms a large, cation permeable pore. When acting via ADGRL1, αLTX activates both pore-mediated and receptor signaling-mediated Ca^2+^ release mechanisms. When acting via ΔLPH, αLTX only induces the pore-mediated mechanisms, and they lead to a slow Ca^2+^ release based on a previously uncharacterized pathway.

### 2.5. α. LTX Releases Ca^2+^ from Non-ER Stores in Model Cells

**Figure 6:**
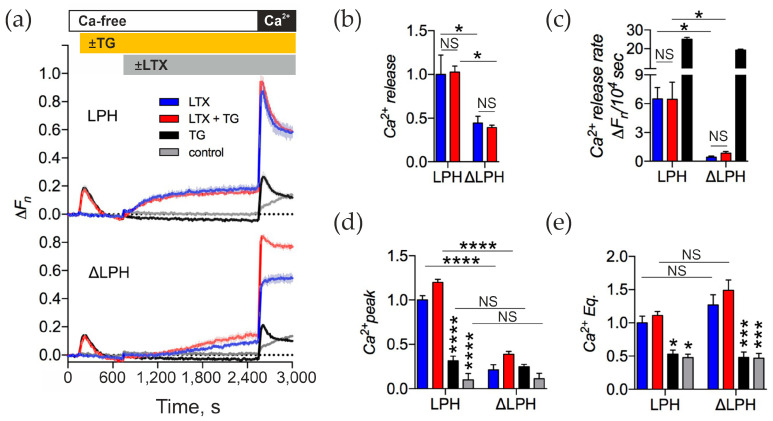
In model cells, αLTX does not release Ca^2+^ from the ER. (**a**) LPH- and ΔLPH-expressing NB2a cells were treated with 0.3 μM TG, then stimulated with 1 nM αLTX and exposed to 2 mM Ca^2+^_e_. Representative fluorescence traces show the averages of three replicates. (**b**–**e**) Ca^2+^_cyt_ changes relative to the αLTX-induced effects in the LPH-expressing cells (blue bars). (**b**) Amplitudes of αLTX-mediated Ca^2+^ release. (**c**) Rates of Ca^2+^ release during the Ca^2+^-free period. (**d**) Amplitudes of transient Ca^2+^ influx in the presence of Ca^2+^_e_. (**e**) Levels of Ca^2+^_e_ Eq. Asterisks show statistical significance compared to LPH-cells + αLTX (blue bars), other comparisons are shown by horizontal lines. Bars are the means ± *SEM* (*n* = 3–4); *, *p* < 0.05; ***, *p* < 0.001; ****, *p* < 0.0001; *NS*, non-significant.

As we showed above, the αLTX-triggered release of neurotransmitters from motor neurons in a Ca^2+^_e_-free medium requires release of Ca^2+^, which at least in part comes from the ER (Section 2.2). Therefore, we investigated whether αLTX also triggers the release of Ca^2+^ from the ER in our model cells expressing the two receptor variants. LPH- and ΔLPH-expressing NB2a cells were treated with 0.3 μM TG in a Ca^2+^-free medium and then stimulated with αLTX (Figure 6).

In Ca^2+^-free media, TG induced the release of Ca^2+^ from the ER in all cells, wild-type or transfected with any ADGRL1 construct (Figure 6a, black lines). This increase in Ca^2+^_cyt_ level developed quickly and returned to baseline levels within 10 min, as Ca^2+^ was quickly extruded from the cytosol by various mechanisms (see Section 3). The depletion of the ER of Ca^2+^ caused the opening of the SOCCs in the plasma membrane, which was revealed as a clear transient peak of Ca^2+^_cyt_ upon the addition of Ca^2+^ to the medium (Figure 6a,d).

Likewise, in Ca^2+^-free media, αLTX caused a release of Ca^2+^ from some stores of LPH-expressing cells (Figure 6a, top, blue line). However, the αLTX-induced release was different from that evoked by TG: it developed more slowly (Figure 6c), and the level of Ca^2+^_cyt_ did not decrease due to Ca^2+^ extrusion (Figure 6a). αLTX-evoked release was at least partially mediated by ADGRL1 signaling, because the ΔLPH construct, despite binding more αLTX, only mediated a very slow increase in Ca^2+^_cyt_ that never reached the same level as in LPH-expressing cells (Figure 6a,c, blue). In addition, pretreatment of receptor-expressing cells with TG did not inhibit the αLTX-induced Ca^2+^ release in either LPH- or ΔLPH -expressing cells (Figure 6a,b,d), suggesting that in these model cells αLTX mobilizes the Ca^2+^ stores that are different from the ER.

On the other hand, similar to TG, αLTX-induced depletion of a Ca^2+^ store led to the opening of Ca^2+^ channels in the plasma membrane, which were detected as a very large transient peak of Ca^2+^ influx upon reintroduction of Ca^2+^_e_ (Figure 6a,d). This peak of SOCE was much larger than that induced by TG alone and was non-significantly increased by pretreating cells with TG (Figure 6a,d). Furthermore, the αLTX-evoked transient SOCE only appeared in cells expressing the signaling receptor construct (LPH) (Figure 6a,d, blue), while treating the ΔLPH cells with both TG and αLTX only produced a Ca^2+^ peak of the size evoked by TG alone (Figure 6d).

Finally, αLTX brought the ultimate equilibrium level of Ca^2+^_cyt_ to a very high value, much higher than in the case of TG treatment (Figure 6e). Because the αLTX-produced Ca^2+^ Eq was similar in the cells expressing either the signaling or mutant receptor, it was likely caused by the presence of αLTX pores in the plasma membrane, which permitted Ca^2+^ entry that was not easily balanced by Ca^2+^ extrusion mechanisms.

These results suggest that TG-mediated inhibition of the SERCA and αLTX-induced ADGRL1-mediated signaling lead to the depletion of different Ca^2+^ stores in our model cells. The depletion of the αLTX-sensitive stores leads to the opening of a larger pool of SOCCs that indeed includes the SOCCs activated by ER depletion. In addition, in both LPH- and ΔLPH-expressing cells, αLTX actions similarly inhibit the mechanisms of Ca^2+^ extrusion and therefore depend on the toxin pore only.

### 2.6. α. LTX-Mediated Ca^2+^ Release and Sustained Elevated [Ca^2+^]_cyt_ Depends on Na^+^_e_

**Figure 7:**
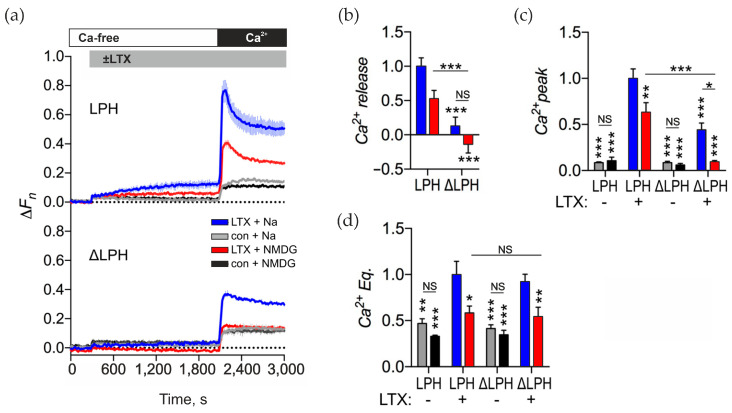
The αLTX pore regulates [Ca^2+^]_cyt_ by inducing Na^+^ influx, while receptor-mediated action does not require Na^+^ influx. (**a**) LPH- and ΔLPH-expressing NB2a cells were incubated in buffer containing Na^+^ or the Na^+^ substitute NMDG, then stimulated with 1 nM αLTX and exposed to 2 mM Ca^2+^_e_. Representative fluorescence traces show the averages of three replicates. (**b**–**e**) Ca^2+^_cyt_ changes relative to the αLTX-induced effects in the LPH-expressing cells in the presence of Na^+^. (**b**) Amplitudes of αLTX-mediated Ca^2+^ release. (**c**) Amplitudes of transient Ca^2+^ influx peaks. (**d**) Levels of Ca^2+^_e_ Eq at the end of experiment. The bars are the means ± *SEM* (*n* = 2–5); the asterisks show statistical significance compared to the LPH/αLTX/Na^+^ condition, other comparisons are shown by horizontal lines. *, *p* < 0.05; **, *p* < 0.01; ***, *p* < 0.001; *NS*, non-significant.

Na^+^_e_ has been shown to contribute to αLTX-mediated exocytosis [45], so we used our model system to test whether Na^+^ influx through αLTX pores actually contributes to [Ca^2+^]_cyt_ regulation. To reveal the role of Na^+^ influx we replaced Na^+^_e_ with N-methyl-D-glucamine (NMDG) (Figure 7a). In untreated control cells, removing Na^+^_e_ had no effect on constitutive Ca^2+^ influx (Figure 7a,c,d). In the absence of Na^+^_e_, αLTX-evoked Ca^2+^ release in LPH-expressing cells was reduced by about 50% (although this change was not statistically significant), while in ΔLPH-expressing cells it was abolished completely (Figure 7a,b, red lines). A similar effect was seen on the transient Ca^2+^ influx peak: without Na^+^_e_, the peak was reduced by 30% in LPH-cells but abolished in ΔLPH-cells. These results clearly indicate that when receptor signaling is blocked and when αLTX solely acts by making membrane pores, both Ca^2+^ release and Ca^2+^ influx entirely depend on the influx of Na^+^_e_ through the toxin pores.

The Ca^2+^ Eq level was reduced in the absence of Na^+^ by the same extent as seen in LPH- and ΔLPH-cells, but was not abolished (Figure 7d), revealing that the elevated equilibrium [Ca^2+^]_cyt_ level was mediated by αLTX pores and not by ADGRL1 signaling, and that high equilibrium [Ca^2+^]_cyt_ is sustained in the αLTX-treated cells due to the influx of Na^+^ and, to a lesser extent, Ca^2+^. These results, together with the lack of decay of [Ca^2+^]_cyt_ released from Ca^2+^ stores, suggest that αLTX inhibits Ca^2+^ extrusion mechanisms by increasing [Na^+^]_cyt_. High Na^+^ is known to reverse the activity of NCX, and this may be the main mechanism of action of αLTX pores.

### 2.7. α. LTX-Mediated [Ca^2+^]_cyt_ Regulation Is Sensitive to SKF-96365

Our observations from Section 2.4 and Section 2.5 indicated that αLTX, by mobilizing Ca^2+^ from intracellular stores, could induce SOCE. Therefore, we tested whether αLTX actions in LPH-expressing cells could be affected by the SOCE inhibitor SKF-96365 (SKF) [46].

**Figure 8:**
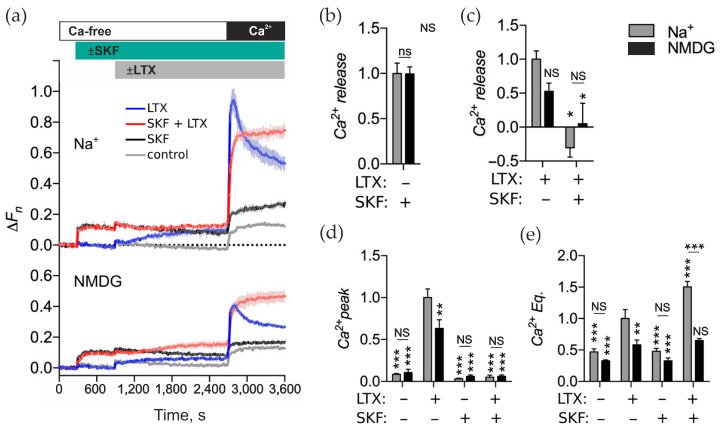
SKF inhibits αLTX-induced Ca^2+^ release and subsequent Ca^2+^ influx. (**a**) LPH-expressing NB2a cells were incubated in buffer containing 145 mM Na^+^ or 145 mM NMDG. The cells were then treated with 100 μM SKF, stimulated with 1 nM αLTX and exposed to 2 mM Ca^2+^_e_. The fluorescence traces are the averages of three replicates and are representative of five independent experiments. (**b**–**e**) Ca^2+^_cyt_ changes relative to the αLTX-induced effects in the LPH-expressing cells in the presence of Na^+^. (**b**) SKF-induced Ca^2+^ release. (**c**) αLTX-mediated Ca^2+^ release with and without prior SKF treatment. (**d**) Amplitude of the Ca^2+^ peaks under respective conditions. (**e**) Ca^2+^ Eq levels after respective treatments. The bars are the means ± *SEM* (*n* = 5); the asterisks show statistical significance compared to LPH/Na^+^/αLTX, other comparisons are shown by horizontal lines; *, *p* < 0.05; **, *p* < 0.01; ***, *p* < 0.001; *NS*, non-significant.

First, we found that, in the presence of Na^+^, SKF caused fast release of Ca^2+^ from an intracellular store (Figure 8a, top, black curve). The resulting elevated [Ca^2+^]_cyt_ did not decay with time and did not lead to an influx of Ca^2+^ upon Ca^2+^_e_ add-back (Figure 8a,d, gray bars as indicated), indicating that SKF inhibited both Ca^2+^ extrusion and SOCCs.

When αLTX was applied after SKF in the presence of Na^+^, it failed to release Ca^2+^ above the effect of SKF (Figure 8a, top, red curve). Furthermore, the transient peak of Ca^2+^ influx (that typically appears after αLTX action followed by re-addition of Ca^2+^_e_) was totally prevented by SKF. These results suggest that SKF and αLTX act, possibly by different mechanisms, on the same intracellular Ca^2+^ stores, but SKF blocks the transient SOCCs that normally respond to the depletion of this store by αLTX. This confirms our hypothesis that αLTX, by depleting intracellular Ca^2+^ stores, induces SOCE.

Finally, comparing the final Ca^2+^ Eq levels in cells treated with SKF and/or αLTX, we found that αLTX alone increased the final Ca^2+^ equilibrium, while SKF did not. When applied together, SKF and αLTX substantially increased the level of Ca^2+^ Eq compared to αLTX alone. This suggests that (i) as SOCCs are blocked by SKF, αLTX-induced opening of these channels does not contribute to the high final Ca^2+^_cyt_; (ii) SKF further inhibits the extrusion of Ca^2+^_cyt_ that enters after αLTX action.

One of Ca^2+^ extrusion pathways is the activity of NCX, which transports Ca^2+^ from cytosol at the expense of transporting Na^+^ into the cell. SKF is known to reverse NCX [47], leading to the accumulation of Ca^2+^ in the cytoplasm. As we demonstrate above, αLTX probably reverses NCX by over-loading the cytoplasm with Na^+^, whereas the effect of SKF on NCX should not depend on increased [Na^+^]_cyt_. As Ca^2+^_e_ was absent during the Ca^2+^ release phase of our experiments, only the NCX present on the membrane of intracellular Ca^2+^ stores (rather than on the plasma membrane) could lead to the accumulation of Ca^2+^ in the cytosol as a result of NCX reversal by SKF. On the other hand, we showed above (Figure 7) that αLTX actions, at least in part, include the influx of Na^+^ into the cytosol via the pores formed by αLTX in the plasma membrane, and this meant that elevation of Na^+^_cyt_ by αLTX—rather than intracellular signaling—could underlie the toxin-induced reversal of the NCX.

To test this possibility, we treated the LPH-cells with SKF and/or αLTX in the presence of NMDG (Figure 8a, bottom). αLTX alone still caused Ca^2+^ release, albeit to a lesser extent (Figure 8c, black bar), and this action resulted in a typical SOCE (Figure 8a, bottom, blue curve), which was also decreased by ~30% (Figure 8d, black bars). This was consistent with a receptor-mediated signaling cascade leading to the depletion of a Ca^2+^ store and the opening of SOCCs. SKF alone also caused a non-decreasing Ca^2+^ release from intracellular stores, but the drug blocked any subsequent SOCE (Figure 8a, bottom, black curve). When αLTX was applied after SKF (Figure 8a, bottom, red curve), it failed to demonstrate both a statistically significant Ca^2+^ release (Figure 8c, black bar) and any transient SOCC opening (Figure 8d, black bar). The ultimate equilibrium Ca^2+^ level achieved by αLTX, alone or after SKF, was decreased by the removal of Na^+^ (Figure 8e).

These results reveal that one major mechanism of αLTX action is Na^+^ influx, which causes Ca^2+^ to leave NCX-containing intracellular Ca^2+^ stores and prevents Ca^2+^ extrusion out of the cell. A second mechanism of toxin action involves ADGRL1-mediated signaling, which does not depend on Na^+^ influx and induces Ca^2+^ release from intracellular stores, followed by the opening of transient SOCCs.

## 3. Discussion

Among the many different mechanisms of αLTX activity (detailed in the Section 1), its effect on neurotransmitter release in the absence of Ca^2+^_e_ remains enigmatic: ADGRL1 signaling is reported to require Ca^2+^_e_ [19,20], while the role of the toxin pore in Ca^2+^-free media seems to be inconsistent with its known ability to pass Ca^2+^_e_ and thus activate exocytosis [4]. Although Ca^2+^-independent effects of the αLTX pore have been reported, including Na^+^_e_ influx [45,48] and Ca^2+^ release from intracellular stores [31], these effects were thought to contribute little or not at all to the toxin action under Ca^2+^-free conditions. While the combination of multiple αLTX-induced mechanisms makes interpretation of its effects difficult, the use of αLTX under sufficiently discriminating conditions can shed light on novel synaptic mechanisms, as has been shown previously [12]. In this paper, we used a non-signaling mutant of ADGRL1 to delineate some of αLTX’s mechanisms.

Using mouse neuromuscular preparations, we found that the well-known αLTX effect—a dramatic increase in the frequency of spontaneous exocytosis at the NMJ—in fact strictly depends on the presence of Ca^2+^_cyt_, which can only come from intracellular stores (Figure 1). Furthermore, TG, which releases Ca^2+^ from the ER, causes a similar, if somewhat smaller, increase in MEPPs frequency and, by doing so, strongly inhibits the effect of αLTX (Figure 2). These results indicate that the enigmatic Ca^2+^_e_-independent αLTX action is most likely based on the canonical mechanism of exocytosis that requires an increase in Ca^2+^_cyt_ to cause vesicle fusion with the plasma membrane. However, the exact pathways by which αLTX induces Ca^2+^ release and the identity of the stores affected by the different αLTX actions remain to be uncovered.

**Figure 9:**
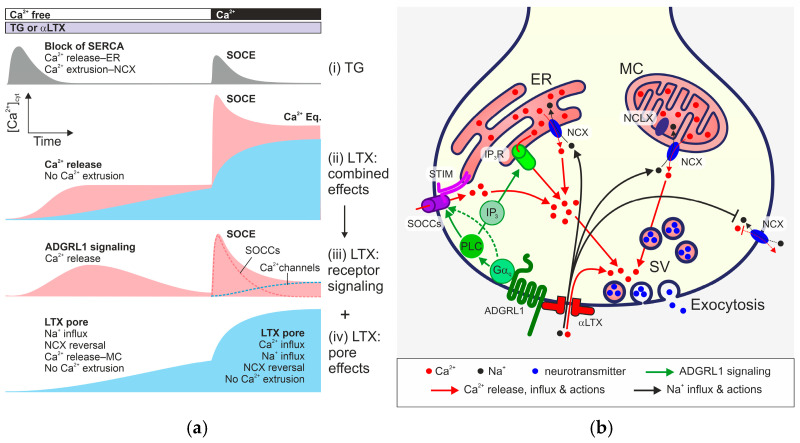
Dual actions of αLTX on Ca^2+^_cyt_ in presynaptic nerve terminals that lead to neurotransmitter release. (**a**) Idealized Ca^2+^_cyt_ dynamics induced by TG and the two LTX actions (after subtracting control traces). (i) TG blocks SERCA and causes Ca^2+^ release from the ER, which activates a transient SOCE upon reintroduction of Ca^2+^_e_; Ca^2+^_cyt_ decays due to NCX activity. (ii) Combined αLTX activity, consisting of the ADGRL1- and pore-mediated effects. (iii) Receptor-dependent αLTX action calculated by subtracting the αLTX pore-mediated effect (iv) from the combined αLTX actions (ii). Receptor-mediated signaling causes a slow release of Ca^2+^ from intracellular stores and subsequent opening of a large pool of SOCCs. Reintroduction of Ca^2+^_e_ leads to a transient SOCE that exceeds that caused by TG. ADGRL1 signaling also opens non-inactivating Ca^2+^ channels that contribute to the elevated Ca^2+^_cyt_ after SOCE. (iv) αLTX pore-mediated effects based on our experiments with ΔLPH and NMDG. αLTX pores mediate the influx of Na^+^_e_, which reverses NCX. Reversal of NCX located on the Ca^2+^ stores (ER and MC) and cell membrane leads to a slow increase in [Ca^2+^]_cyt_, and inhibits Ca^2+^_cyt_ extrusion. Upon reintroduction of Ca^2+^_e_, Ca^2+^_e_ influx via αLTX pores and inhibition of Ca^2+^_cyt_ extrusion elevate the [Ca^2+^]_cyt_ further. (**b**) A model of αLTX action. αLTX binds and activates ADGRL1, and forms pores in the cell membrane. Na^+^ and Ca^2+^ enter though the αLTX pores. Elevated [Na^+^]_cyt_ reverses NCX located on the MC, ER, and cell membrane. This releases Ca^2+^ from the MC and ER, and inhibits Ca^2+^_cyt_ extrusion. The pore-mediated [Ca^2+^]_cyt_ increase triggers exocytosis of synaptic vesicles (SV). αLTX also activates G protein signaling via ADGRL1, resulting in Ca^2+^ release from the ER and/or other stores, and the activation of SOCCs (via store depletion and/or by direct signaling). However, ADGRL1-mediated αLTX action requires Ca^2+^ influx via SOCCs to develop its full effect and cause a burst-like release of SVs. IP_3_, inositol 1,4,5-trisphosphate; IP_3_R, IP_3_ receptor; NCLX, Na^+^-Ca^2+^ exchanger of the internal MC membrane; STIM, proteins detecting Ca^2+^ release from ER; SV, synaptic vesicles.

To answer this question, we first compared the effects of αLTX and TG in neuroblastoma cells, used here as a model. In these and most other cells, TG depletes the ER of Ca^2+^, and the depletion of the Ca^2+^ store stimulates the opening of SOCCs on the plasma membrane, which manifests itself as a transient SOCE upon reintroduction of Ca^2+^ (Figure 9(ai)). We found that αLTX also releases Ca^2+^ from intracellular stores in these cells but only if they are stably expressing αLTX receptors (Figure 4). To dissect the receptor-mediated and pore-mediated αLTX actions, we employed signaling (LPH) and non-signaling (ΔLPH) receptor constructs (Figure 5). By subtracting the pore effects, which develop when αLTX binds ΔLPH (Figure 9(aiv)), from the overall αLTX effects in LPH-expressing cells (Figure 9(aii)), we identified the receptor-mediated actions of αLTX (Figure 9(aiii)).

Acting via receptor signaling, αLTX causes a relatively slow release of Ca^2+^ from intracellular stores (Figure 4; Figure 9(aiii)), which to a small extent depends on the presence of Na^+^_e_ (Figure 7). This effect is transient and is not attenuated by pretreatment with TG (Figure 6), indicating that, in these model cells, ADGRL1 signaling mobilizes a Ca^2+^ store that is different from the ER, whose role is critical for αLTX effects on the NMJ. This highlights the limited applicability of neuroblastoma cells to studying presynaptic mechanisms. The depletion of this store also leads to the opening of a pool of SOCCs, revealed upon reintroduction of Ca^2+^_e_, which is larger than the TG-sensitive pool of SOCCs (Figure 3, Figure 4, Figure 5 and Figure 6; Figure 9(aiii)). This peak of Ca^2+^ entry only occurs in cells possessing a signaling ADGRL1. The SOCC inhibitor SKF blocks these channels (Figure 8). The nature of the αLTX receptor-sensitive Ca^2+^ store is not known but it could include MC, as previously suggested by Tsang et al. [31]. Likewise, the ADGRL1 signaling mechanisms and the identity of the SOCCs sensitive to receptor signaling remain to be further investigated; such a study should be conducted in neurons, which constitute the natural target of αLTX, and employ αLTX mutants that lack the ability to form pores.

The αLTX pore action in the model cells appears to be more evident: our data indicate that a major function of the pore both in the absence of Ca^2+^ and, to a large extent, in its presence, is to mediate Na^+^ rather than Ca^2+^ influx. Loading cells with Na^+^ is known to reverse the direction of NCX, which is located in organelles (MC and ER) and cell membranes [35,36,49,50]. NCX reversal leads to both the leak of Ca^2+^ from organelles and the inhibition of its extrusion from the cytosol, where it slowly accumulates with time in contrast to TG-induced Ca^2+^_cyt_ (Figure 6 and Figure 7; Figure 9(ai)). Our studies with SKF support this, because this SOCE inhibitor [24,46] also reverses NCX [47]. Upon Ca^2+^_e_ reintroduction, the αLTX pore elicits an increase in Ca^2+^_cyt_, which lacks the transient peak of Ca^2+^ influx and is maintained due to (i) the influx of Ca^2+^ and Na^+^ through the αLTX pores and (ii) the inhibition of Ca^2+^_cyt_ extrusion by high Na^+^_cyt_.

Previously, Tsang et al. [31] found that in 0 Ca^2+^_e_, BAPTA-AM blocked the αLTX-induced increase in [Ca^2+^]_cyt_ in frog NMJs but did not inhibit the effect of αLTX on the frequency of MEPPs. These results are in disagreement with our observations (Section 2.1), which irrefutably show an inhibitory effect of BAPTA on the αLTX-evoked release of ACh at mouse NMJs. One possible reason could be the difference between the frog and mouse nerve terminals: frog NMJs are much larger and contain many times more vesicles and other intracellular organelles than mouse NMJs. It is possible that intracellular BAPTA could block the global Ca^2+^_e_ build-up in the large frog NMJs but still allow Ca^2+^ action near the vesicle release sites, as suggested previously [48]. Another possible reason for the disagreement between their optical and electrophysiological recordings could be that BAPTA-AM precipitation, as we describe above, could (i) produce an insufficient concentration of intrasynaptic BAPTA, especially in the NMJs located deeper in the muscle and (ii) optically obscure the Ca^2+^ fluorescence signal. Indeed, we see that BAPTA-AM produces high turbidity in bath solution and while it blocks all exocytosis in superficial NMJs, it is ineffective in deeper synapses that respond to αLTX (Figure 1).

Also, Tsang et al. [31] observed very little effect of TG on spontaneous MEPP frequency and no effect on the excitatory action of αLTX. This, to some extent, agrees with our data: while ER depletion inhibits, albeit incompletely, the αLTX-induced exocytosis in mouse NMJs (Figure 2), it does not affect the αLTX-induced Ca^2+^ release in model neuroblastoma cells (Figure 6). On the other hand, TG itself induces a very large increase in MEPP frequency in mouse NMJs at 0 Ca^2+^_e_ (Figure 2). Again, the likely reason for this discrepancy is the architecture of the cells studied: both frog NMJs and ADGRL1-transfected cells have a much larger volume than mouse NMJs. The lack of TG effect on the αLTX action in these large cells could be due to the large buffering capability of their MC [50] and to the distinct distribution of organelles within the cell. Thus, the bulk of Ca^2+^ that is indirectly and relatively slowly released from the ER by TG or by ADGRL1-mediated signaling could be taken up by MC in neuroblastoma cells and frog NMJs but not in smaller mouse NMJs. Also, it is important to stress that our results clearly demonstrate that, at least in the model cells, there are two distinct sources of Ca^2+^ which are depleted by different mechanisms (Section 2.5). The nature of these stores awaits proper elucidation.

As a final note, our observations underscore the importance of selecting adequately reduced systems to investigate complex biological phenomena, such as the complex effects of a toxin on an equally complex mechanism of neurotransmitter exocytosis. Further work will be required to dissect the receptor-mediated actions of αLTX, identify the SOCCs involved and delineate the role of different ADGRL homologs in the toxin’s effect.

## 4. Conclusions

Wild-type αLTX strictly requires Ca^2+^_cyt_ to produce massive neurotransmitter exocytosis in the absence of Ca^2+^_e_. Under these conditions, the toxin induces the depletion of distinct Ca^2+^ stores in different cells and thus elevates [Ca^2+^]_cyt_ by at least two separate mechanisms: receptor signaling and the influx of Na^+^ through αLTX pores. ADGRL1-mediated depletion of the ER and/or other stores activates transient Ca^2+^ channels (SOCCs) that are independent of the αLTX pore. The toxin pore mediates an increase in [Na^+^]_cyt_ that causes NCX reversal and leads to release of Ca^2+^ from the ER and/or MC and inhibits Ca^2+^_cyt_ extrusion, providing the source of Ca^2+^_cyt_ for exocytosis via classical Ca^2+^-induced membrane fusion.

## 5. Materials and Methods

### 5.1. Materials

All chemicals and reagents were purchased from Sigma-Aldrich (Merck Life Science UK Limited, Gillingham, Dorset, UK) unless otherwise stated. The following antibodies were used for Western blotting: rabbit anti-V5 polyclonal antibody, rabbit anti-αLTX serum (produced in the lab), and IRDye^®^ 800CW goat anti-rabbit IgG secondary antibody (LI-COR Ltd. United Kingdom, Cambridge, UK).

αLTX was purified from lyophilized venom of black widow spiders, *Latrodectus lugubris*, as described previously [51]. Toxin homogeneity was verified by SDS-polyacrylamide gel electrophoresis (SDS-PAGE) and amino acid analysis. The latter method was also employed to determine protein concentration in a reference sample of αLTX, which was subsequently used in conjunction with SDS-PAGE, Coomassie R250 staining and computer-assisted densitometry to quantify all other toxin preparations. To demonstrate the specific action of the purified natural toxin, some experiments were performed using 0.5 nM recombinant αLTX expressed in a baculovirus system and purified by affinity chromatography [19,52], yielding identical results.

A 50 mM stock solution of BAPTA-AM (Thermo-Fisher Scientific, Oxford, UK, Life Technologies Limited, Paisley, UK) was prepared in DMSO, then diluted with a physiological buffer containing 0.1% Pluronic F-127 (Thermo-Fisher Scientific, Oxford, UK) to obtain a 5 mM secondary stock and sonicated.

### 5.2. Neurotransmitter Release

Spontaneous synaptic activity at mouse NMJs (in the form of MEPPs) was recorded using *flexor digitorum brevis* neuromuscular preparations dissected from hind paws of 3–6 weeks-old male mice, pinned inside Petri dishes coated with Sylgard (Dow Silicones UK Ltd., Barry, Wales, UK) and containing preformed perfusion chambers. The preparation was observed under a high-power binocular microscope with dark-field illumination and perfused with oxygenated physiological buffer containing (in mM): NaCl, 137; KCl, 5; MgCl_2_, 1; EGTA, 0.2; glucose, 5.6; HEPES, 10; pH 7.5). When required, the perfusion was stopped, and 0.5 nM αLTX, 200–500 μM BAPTA-AM, 10 μM TG, 20 mM KCl and/or 2.2 mM CaCl_2_ was added (final concentrations). Changes in the postsynaptic membrane potential (*V*m) were detected using sharp glass microelectrodes filled with 5 M ammonium acetate (impedance ~70 MOhm), pre-amplified using an Axoclamp 2B amplifier (Molecular Devices, LLC, San Jose, CA, USA) in the current clamp mode, amplified and filtered using a differential amplifier with a high-frequency filter (LPF202A, Warner Instruments) and a harmonic frequency quencher (HumBug, Digitimer Ltd., Welwyn Garden City, Hertfordshire, UK), and then digitized with a Digidata 1322A digitizer controlled by the AxoScope 10.7 software (Molecular Devices). The traces were analyzed using the Mini Analysis software Version 6.0.7 (Synaptosoft Inc., Decatur, GA, USA).

### 5.3. Cell Culture

NB2a cell lines stably transfected with LPH, and ΔLPH constructs were kindly provided by K. E. Volynski. The cells were cultured in complete medium (Dulbecco-modified Eagle’s medium containing 0.5 mM GlutaMAX^TM^ and 10% fetal bovine serum). Cells were kept at 37 °C in a humidified atmosphere consisting of 5% CO_2_. Cells were allowed to grow to 80% confluency before passaging, and cells were detached using 0.05% Trypsin-EDTA. Differentiation was induced 24 h after plating cells by replacing complete medium with Neurobasal-A medium supplemented with 2% B-27 and 0.5 mM GlutaMAX^TM^, and experiments were performed 24–48 h after differentiation was induced.

### 5.4. ADGRL1 Construct Expression Assay

Cells seeded in T25 tissue culture flasks were differentiated for 40–44 h and then detached by tapping the flask. Cells were resuspended in PBS containing 1 mg/mL BSA at a concentration of 5 × 106 cells per mL. Cells were centrifuged and lysed in PBS containing 1% Triton X-100 for 30 min, on ice. A loading buffer (final concentrations were: 0.0625 M Tris (pH 6.8), 2% SDS, 10% glycerol, 0.1 M DTT, 0.01% bromophenol blue) was added to samples, vortexed, heated at 50 °C for 20 min and stored at –20 °C.

### 5.5. α. LTX Binding Assay

Cells seeded in T25 flasks were differentiated for 40–44 h and then detached by tapping the flask. Cells were resuspended in PBS containing 1 mg/mL BSA at a concentration of 5 × 10^6^ cells per mL and transferred to 1.5 mL tubes. Cells were exposed to 5 nM αLTX for 10 min on ice, then spun down at 40× *g* for 2 min. The supernatant, which contained unbound αLTX, was transferred to fresh tubes. The cell pellet was washed briefly with PBS, spun down for 10 s, and then lysed in PBS containing 1% Triton X-100 for 30 min on ice. Cells were spun down at 12,000× *g* for 20 min at 4 °C and the supernatant, which contained αLTX bound to ADGRL1, was transferred to fresh tubes. Loading buffer was added to samples, vortexed, heated at 50 °C for 20 min and stored at −20 °C.

### 5.6. Western Blotting

Whole-cell lysates and supernatants were separated by SDS-PAGE. Polyacrylamide gels (8%) were prepared, using 40% ProtoGel, ProtoGel Stacking Buffer, 4X ProtoGel Resolving Buffer, TEMED and ammonium persulfate (National Diagnostic, Atlanta, GA, USA)according to manufacturer’s protocol. The inner and outer chambers of the gel tank were filled with TRIS/Glycine/SDS running buffer (National Diagnostics, Atlanta, GA, USA). The samples (30 μL) and molecular weight markers (PageRuler, Thermo-Fisher Scientific, Oxford, UK) were separated by running 120 V through the gel for up to 1.5 h. Proteins were electrophoretically transferred onto polyvinylidene fluoride membranes (0.45 μm pore) in TRIS/Glycine transfer buffer containing 20% methanol (National Diagnostics), at a constant current of 180 mA for 1 h.

Membranes blocked in 5% non-fat milk dissolved in Tris-buffered saline with Tween-20 (TBST, National Diagnostics) for 1 h at RT and stained using respective primary antibodies (1:1000 dilution) and fluorescent secondary antibody (1:2000 dilution). Fluorescent detection was performed using Odyssey imaging system (LI-COR Ltd. United Kingdom) and protein bands were analyzed in ImageJ (version 1.45m; National Institutes of Health, Madison, WI, USA; doi:10.1038/nmeth.2089).

### 5.7. Ca^2+^_cyt_ Recordings

NB2a cells were seeded onto black-walled, clear-bottomed 96-well plates (VWR International, LLC, Radnor, PA, USA) and differentiated for 24–48 h. Cells were loaded with the cell-permeable Ca^2+^ indicator Fluo-4-AM (Thermo-Fisher Scientific, Oxford, UK, Life Technologies Limited, Paisley, UK) according to manufacturer’s protocol, and fluorescence was detected on a Fluoroskan Ascent FL microplate fluorometer (Labsystems Diagnostics Oy, Helsinki, Finland) with 495/538 nm excitation/emission filters. Fluorescence was measured in multiple replicates every 15 s with 100 ms integration time. Cells were equilibrated in Recording buffer (in mM: NaCl, 145; KCl, 5.6; glucose, 5.6; MgCl_2_, 1; HEPES, 15; BSA, 0.5 mg/mL; sulfinpyrazone, 0.25; pH, 7.4). The experimental protocols are described in the Results. αLTX and pharmacological inhibitors (TG, SKF) were added to individual wells by pipette during a brief pause in recording, while buffer containing Ca^2+^ (to achieve a final concentration of 2 mM) was added automatically via an internal Fluoroskan dispenser. Experiments were usually performed in triplicate and repeated independently at least three times.

As Fluo-4 is a single-wavelength, non-ratiometric indicator, its fluorescence had to be compensated for the variance in the number of cells scanned in each well. Therefore, Equation 1 was used to normalize fluorescence intensity (*F*) to an average initial baseline value (*F_min_*) and a maximal value (*F_max_*), obtained by permeabilizing cells with 1% Triton X-100 at the end of each experiment.

(1) $$∆F_{n}=\frac{F-F_{min}}{F_{max}-F_{min}}$$

Release of Ca^2+^ from intracellular stores was measured as the increase in ∆*F_n_* from the baseline. Ca^2+^_cyt_ equilibrium in the presence of Ca^2+^_e_ (Ca^2+^ Eq) was measured as ∆*F_n_* amplitude and the transient Ca^2+^ peak was measured as the ∆*F_n_* amplitude above Ca^2+^ Eq.

### 5.8. Statistical Analysis

Statistical analysis was performed in Prism 6 software (GraphPad Software, Boston, MA, USA). Unless otherwise stated, two-tailed Student’s *t*-test was performed for comparisons between two groups, or one-way analysis of variance (ANOVA) was used for three or more groups with Bonferroni correction. Statistical significance was accepted at *p* < 0.05. The level of significance was also indicated on graphs (*p* < 0.05:*, *p* < 0.01:**, *p* < 0.001:***, *p* < 0.0001:****).

## Data Availability

The data supporting reported results can be found in the following publicly archived datasets: https://figshare.com; 10.6084/m9.figshare.28138064.

## Supplementary Materials

The following supporting information can be downloaded at: https://www.mdpi.com/article/10.3390/toxins17020073/s1, Figure S1: 200 μM BAPTA-AM does not produce a sufficient cytosolic level of BAPTA to chelate all Ca^2+^_e_ entering via voltage-gated Ca^2+^ channels. (**a**) An example of the effect of 20 mM KCl on the frequency of spontaneous MEPPs in mouse neuromuscular preparations in the presence of 2 mM Ca^2+^_e_, continuously recorded from an individual muscle fiber. (**b**) Top, the experimental protocol: initial incubation in a Ca^2+^-free buffer; treatment with 200 μM BAPTA-AM; an extended washing step with a Ca^2+^-free buffer; addition of 20 mM KCl/2 mM Ca^2+^_e_. Bottom, changes in the frequency of MEPPs under the experimental conditions indicated above. (**c**,**d**) Mean MEPP frequencies and amplitudes during respective experimental stages. The bars are the means ± *SEM*; the bar colors correspond to protocol phases; asterisks show statistical significance compared to Ca^2+^_e_-free control, unless indicated by lines; *, *p* < 0.05; **, *p* < 0.01; ***, *p* < 0.001; *NS*, non-significant; for each condition shown the *n* = 6–15 individual muscle fibers from 3 independent neuromuscular preparations.
