# Supplementary material for: α-Latrotoxin Actions in the Absence of Extracellular Ca2+ Require Release of Stored Ca2+
Source: Toxins (Basel). 2025 Feb 6;17(2):73. doi: 10.3390/toxins17020073 (PMC11860464; doi:10.3390/toxins17020073)
Supplement: Supplementary file 1 [file toxins-17-00073-s001.zip › toxins-3438470-supplementary.pdf]

Supplementary Information

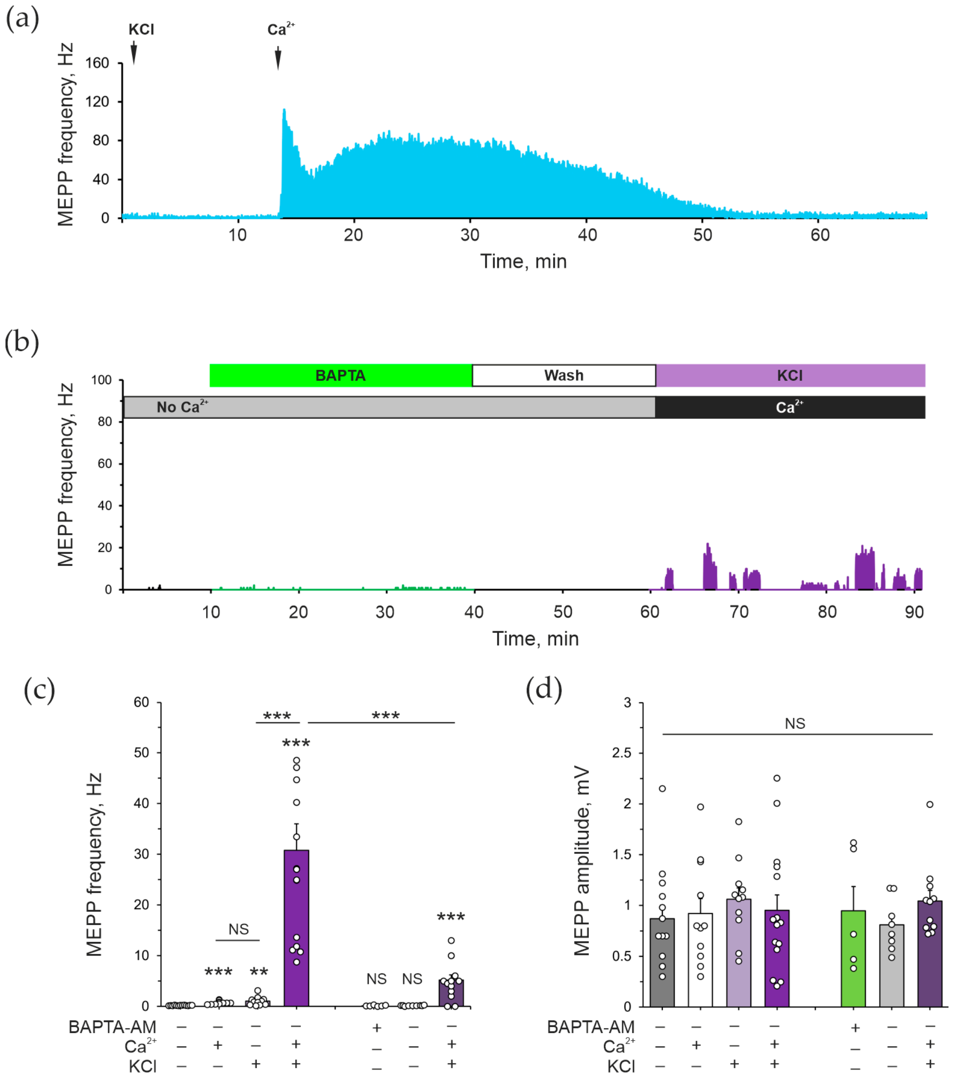

Figure S1. 200 μM BAPTA-AM does not produce a sufficient cytosolic level of BAPTA to chelate all Ca^{2+}_{e} entering via voltage-gated Ca^{2+} channels. (**a**) An example of the effect of 20 mM KCl on the frequency of spontaneous MEPPs in mouse neuromuscular preparations in the presence of 2 mM Ca^{2+}_{e}, continuously recorded from an individual muscle fiber. (**b**) Top, the experimental protocol: initial incubation in a Ca^{2+}-free buffer; treatment with 200 μM BAPTA-AM; an extended washing step with a Ca^{2+}-free buffer; addition of 20 mM KCl/2 mM Ca^{2+}_{e}. Bottom, changes in the frequency of MEPPs under the experimental conditions indicated above. (**c, d**) Mean MEPP frequencies and amplitudes during respective experimental stages. The bars are the means ± SEM; asterisks show statistical significance compared to Ca^{2+}_{e}-free control, unless indicated by lines; *, $P < 0.05$; **, $P < 0.01$; ***, $P < 0.001$; *NS*, non-significant; for each condition shown the n = 6–15 individual muscle fibers from 3 independent neuromuscular preparations.
